# Plasma proteins and the risk of inflammatory bowel diseases: A two-sample Mendelian randomization study

**DOI:** 10.1097/MD.0000000000044738

**Published:** 2025-10-10

**Authors:** Zheng Jiao, Ge-Ge Li, Ling-Shuo Bai, Jian-Zeng Guo, Li-Jian Pang

**Affiliations:** a Department of Internal Medicine, Affiliated Hospital of Liaoning University of Traditional Chinese Medicine, Shenyang, China; b Department of College of Health Preservation and Rehabilitation, Liaoning University of Traditional Chinese Medicine, Shenyang, China; c Department of The First Clinical Medical College, Liaoning University of Traditional Chinese Medicine, Shenyang, China; d Department of Institute of Life Sciences, China Medical University, Shenyang, China.

**Keywords:** genetic association, histotype, inflammatory bowel disease, Mendelian randomization, plasma proteins

## Abstract

Dysregulated plasma proteins are therapeutic targets for inflammatory bowel disease (IBD), which is a major global health burden. There is limited causal evidence for these associations. Mendelian randomization (MR) was applied to assess the genetic links between plasma proteins and IBD risk. Genetic instruments for 3282 plasma protein phenotypes were derived from genome-wide association studies (GWAS). Summary statistics from IBD GWAS (overall, Crohn disease [CD] and ulcerative colitis [UC]) were analyzed via inverse-variance weighted (IVW) regression, estimating odds ratios (OR) and 95% confidence intervals (CI). Four proteins showed associations with overall IBD risk: erythrocyte band 7 integral membrane protein (OR = 0.84, 95% CI = 0.77–0.90), Interleukin-1 receptor-like 1 (IL1RL1; OR = 1.07, 1.04–1.10), Interleukin-18 receptor 1 (IL18R1; OR = 1.08, 1.04–1.12), and alcohol dehydrogenase 1 B (ADH1B; OR = 0.85, 0.79–0.91). Subtype analyses revealed CD-specific associations with erythrocyte band 7 (OR = 0.82, 0.76–0.90) and IL1RL1 (OR = 1.10, 1.05–1.14), while UC risk correlated with ADH1B (OR = 0.85, 0.78–0.91) and high-affinity immunoglobulin gamma Fc receptor I (FCGR1A; OR = 0.82, 0.75–0.90). This MR study identified causal associations between specific plasma proteins (erythrocyte band 7, IL1RL1, IL18R1, ADH1B, and FCGR1A) and IBD pathogenesis, with distinct molecular profiles for CD and UC. These findings implicate dysregulated innate immunity and metabolic pathways, offering mechanistic insights into therapeutic development. Further validation of protein-mediated mechanisms is warranted.

## 1. Introduction

Inflammatory bowel diseases are divided into ulcerative colitis (UC) and Crohn disease (CD). CD is similar to UC, both of which have been classified as chronic IBD and cause digestive disorders and inflammation of the gastrointestinal tract.^[1]^ IBD results from an interaction between genetic and environmental factors. An epidemiological report from Western industrialized countries showed that the incidence of IBD was 0.5% in 2010, 0.75% in 2020, and 1.0% in 2030.^[2]^ With the prolongation of the disease course, IBD will develop a variety of complications, seriously affecting the quality of life and prognosis of patients, and has become one of the major health problems in the world.^[3]^ Therefore, there is an urgent need to develop effective preventive strategies to reduce the public health burden of IBD.

Human blood, an abundant and accessible biological specimen, serves as a valuable resource for assessing individual and population health. Following standardized separation, plasma – the acellular component – contains a diverse array of circulating proteins originating from tissues, organs, and systemic circulation.^[4]^ These plasma proteins are integral to critical biological functions including signal transduction, transport, growth regulation, and host defense, while concurrently reflecting the functional status of the circulatory system and associated tissues^[4,5]^ In the specific context of IBD, experimental UC models demonstrate upregulation of the CXCR2/ELR⁺ CXC chemokine axis (including CXCL3/6), promoting neutrophil-dependent tissue damage. Concurrently, induced growth factors (TGFβ, PDGF, VEGF) facilitate mucosal healing and epithelial regeneration.^[6–8]^ The SomaScan Assay utilizes nucleic acid aptamers for high-throughput plasma protein profiling, enabling large-scale biomarker discovery.^[9]^ Current platforms quantify approximately 10% of the human proteome, facilitating comprehensive investigation of plasma protein-disease associations. This study employs genetic epidemiology to investigate causal relationships between circulating proteins and IBD subtypes.

Randomized controlled trials are the gold standard for inferring causality in conventional epidemiological research.^[10]^ However, due to the strict and difficult conditions of trial design and implementation, there is a need to consider ethical medical issues. Therefore, it is often difficult to directly explore the causal relationships between biomarkers and diseases.^[11]^ Since its inception as a genetic epidemiological approach, Mendelian randomization (MR) has been widely used as a powerful tool to infer causality from observational data. At its core, MR leverages the natural, random assortment of genetic variants (like SNPs) during conception, which mimics the random assignment in an randomized controlled trials (RCT). Individuals inherit genetic variants randomly from their parents, making these variants largely independent of environmental and lifestyle factors that typically confound observational studies. MR uses these genetic variants as proxies (or “instrumental variables”) for a modifiable exposure of interest (e.g., levels of a specific plasma protein).^[12]^ By examining the association between these genetic proxies and the outcome (e.g., IBD risk), MR can provide evidence about whether the exposure causally influences the outcome, under key assumptions that the genetic variants are robustly associated with the exposure and not linked to the outcome through pathways other than the exposure.^[13]^ Crucially for chronic diseases like IBD, which have long latent periods and complex etiologies, MR offers a unique opportunity to investigate causal risk factors and biomarkers without the biases introduced by reverse causation (where disease status influences biomarker levels) or confounding by lifestyle factors that accumulate over the disease course.^[14]^ Using this feature, based on MR studies, the information of single-nucleotide polymorphisms (SNP) was identified and extracted from the level of genome-wide significance, and these SNPs were used as instrumental variables (IVs) to assess the relationship between exposure and outcome allowing estimation of an odds ratio (OR) for the outcome per unit change in the exposure driven by the genetic instrument and mean differences where applicable.^[15]^ In the context of IBD research, identifying circulating proteins with causal roles in disease pathogenesis via MR can directly inform therapeutic target prioritization. Such causal proteins represent high-confidence candidates for drug development, as modulating their activity may alter disease risk or progression. Consequently, MR findings hold significant promise for accelerating the discovery of novel therapeutic strategies for IBD.

## 2. Materials and methods

### 2.1. Study design

**Figure 1.**
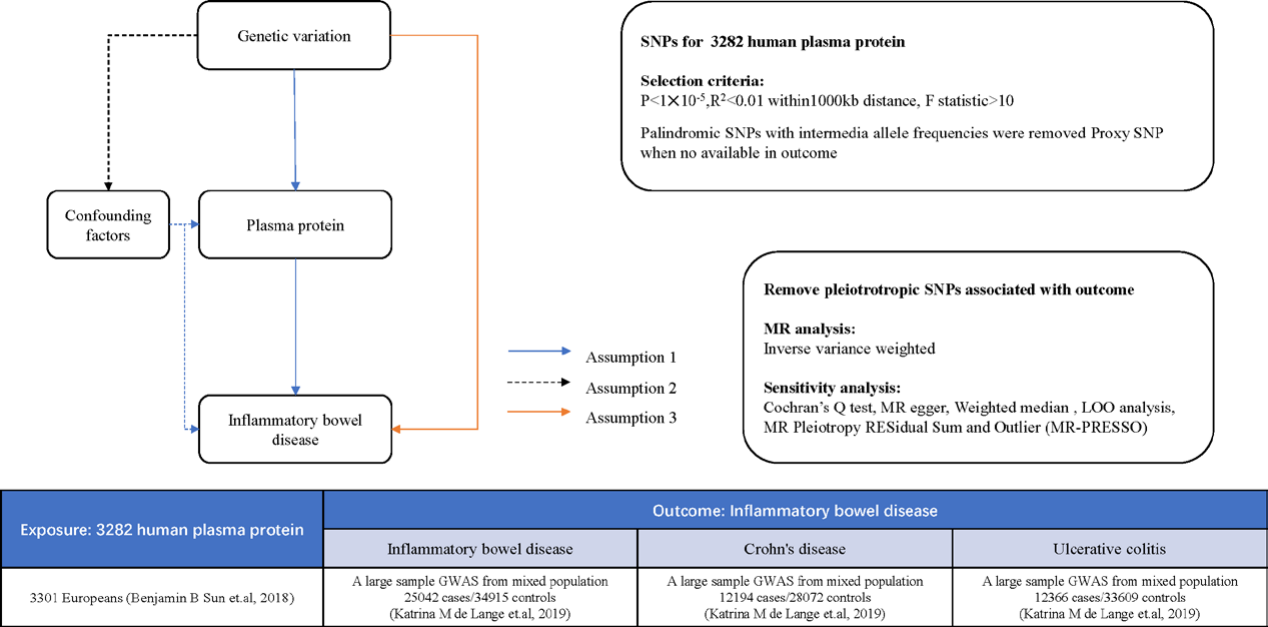
Overall study design based on Mendelian randomization. MR concepts and underlying IV assumptions are shown in a directed acyclic graph (I–III). The data sources and statistical methods used in the study are presented in the table. MR = Mendelian randomization, IV = instrumental variable.

MR studies utilize genetic variants that are substantially correlated with exposure as risk factors, known as IVs, to assess the causation between exposure and outcomes. The MR methodology reduces residual confounding and reverse causation, thereby improving the causal inference of exposure-outcome relationships. This arises from the notion that the genetic variants employed to examine modified exposure effects are randomly assigned at conception, and are thus unaffected by environmental influences or susceptible to reverse causation. Genetic variations pertaining to the risk of 3282 human plasma proteins and IBD and its histotypes (UC and CD) were sourced from published genome-wide association studies (GWAS). We conducted a comprehensive evaluation of the causal effects of 3282 human plasma proteins on IBD and its histotype (UC and CD) risk using a two-sample MR approach (Fig. 1).

### 2.2. Data sources

SNPs of 3282 human plasma proteins and IBD and its histotypes (UC and CD) were screened from a previous GWAS. To prevent potential bias from consanguineous cases, the individuals included in this study of human plasma proteins were of European ancestry. To study IBD and its histotypes more widely, individuals with IBD and its histotypes were included in this study from mixed ancestry, taking into account the number of patients and controls. For the analysis of 3282 human plasma proteins, genetic variation data from 3301 Europeans were utilized, ensuring no overlapping cohorts. In brief, modified single-stranded DNA SOMAmers were used to bind specific protein targets, which were then quantified using a DNA microarray. Protein concentrations were quantified as relative fluorescent units. The genetic variation associated with human plasma proteins was derived from summary data sourced from the Division of Cardiovascular Medicine, Addenbrooke’s Hospital (University of Cambridge), and can be accessed on the website https://gwas.mrcieu.ac.uk/datasets/, with registration numbers ranging from prot-a-1 to prot-a-3282. This study encompassed 1927 genetic associations with 1478 proteins, including trans-associations for 1104 proteins.^[16]^ The outcome data for IBD and its histotypes (UC and CD) were obtained from large-scale GWAS statistics conducted by the Wellcome Trust Sanger Institute, involving 25,305 individuals, and meta-analyzed with published summary statistics, yielding a total sample size of 59,957 subjects, which aimed to identify risk loci associated with IBD diagnosed using accepted endoscopic, histopathological, and radiological criteria. By subtype, 12,194 cases and 28,072 controls were used for the outcome of CD, and 12,366 cases and 33,609 controls were used for the outcome of CD. The outcome data for IBD and its histotypes (UC and CD) can also be accessed at https://gwas.mrcieu.ac.uk/datasets/, with registration numbers ranging from ebi-a-GCST004131 to ebi-a-GCST004133.^[17]^

### 2.3. The selection of instrument variables

To ensure the validity of our causal inference, we imposed 3 crucial assumptions on the eligible IVs: first, the IVs (SNPs being utilized in this case) must exhibit a clear and quantifiable link to the exposure(s) under investigation. Second, these IVs should not be correlated with confounding variables. Lastly, IVs should solely influence outcomes through the exposure (s) being studied. To identify independent IVs with substantial impact, we used R software (Version 4.3.2) and set a genome-wide significance *P*-value threshold to screen SNPs that can be IVs. SNPs associated with plasma proteins at genome-wide significance (*P* < 5 × 10^−8^) were extracted as candidate IVs. Given that the significant SNPS extracted from most proteins are insufficient (n < 3), the threshold is relaxed to *P* < 1 × 10^−5^ to ensure the strength of the instrument.^[18]^ To maintain the independence of the genetic variants, independent SNPs were retained using PLINK’s clumping algorithm (linkage disequilibrium threshold: *r*² < 0.01 within 10,000 kb windows) based on the 1000 Genomes European reference panel. SNPs palindromic or with ambiguous strand orientation were discarded to prevent allele mismatch. Additionally, *F*-statistics were calculated for each protein-exposure pair. IVs with *F* < 10 were excluded to mitigate weak instrument bias. Harmonization of exposure-outcome datasets was performed by exact matching of SNP identifiers (rsID), chromosomal positions, and effect alleles.^[19]^ During this process, variants absent in either dataset were systematically excluded using the TwoSampleMR package. No data imputation was applied; analyses were restricted to SNPs concurrently available in both GWAS sources.

### 2.4. Statistical analysis

The primary method used to assess the causal relationship between exposure and outcome in the MR analysis was inverse variance weighting (IVW).^[20]^ Actually, IVW is a meta-analysis technique, which integrates the Wald ratio of individual IVs under a multiplicative random effects model and is recognized as a cornerstone in two-sample MR analysis.^[21]^ The ORs were used to quantify the levels of incremental risk factors per standard deviation. To reinforce our research findings, we employed the MR-Egger and weighted median methods as complementary approaches.

Cochran *Q* test was applied within the IVW framework to evaluate the heterogeneity of the causal estimates across SNPs.^[22]^ Furthermore, we conducted an analysis of leave-one-out sensitivity to assess the impact of individual SNPs on the overall MR estimate. Although the MR-Egger method is useful, it must yield imprecise and non-statistically significant results when dealing with a limited number of SNPs. This method assumes independence between instrumental strength and direct effect.^[21]^ If the intercept significantly deviated from zero, horizontal pleiotropy was indicated and MR-Pleiotropy residual Sum and Outlier (MR-PRESSO) was further employed to detect horizontal multidirectional outliers, and an analysis was conducted by excluding these outliers.

For human plasma protein traits, the correction of the false discovery rate was applied to account for multiple testing with *P*-adjust <.1, which indicates statistical significance. We also visually assessed symmetry using funnel and scatter plots. The R package “TwoSampleMR” (version 0.5.6) was used to conduct the analyses (R version 4.3.2).

### 2.5. Role of funders

The funders did not play a role in the design of the study; collection, analysis, and interpretation of the data; writing of the manuscript; or the decision to submit the manuscript for publication.

## 3. Results

### 3.1. Selection of IVs

In the current study, GWAS data of 3282 human plasma proteins were used to screen for IVs. After the initial screening, there were 4 different types of plasma proteins with potential causal relationships with IBD (Table S1, Supplemental Digital Content, https://links.lww.com/MD/Q147). In addition, for the 2 subtypes of IBD, we identified 2 different types of plasma proteins that may be associated with disease risk (Table S2, Supplemental Digital Content, https://links.lww.com/MD/Q147). The F-statistics for all IVs were markedly >10, indicating no evidence of weak instrument bias.

### 3.2. Causal relationships between the plasma proteins phenotypes and risks of IBD and its subtypes

**Figure 2.**
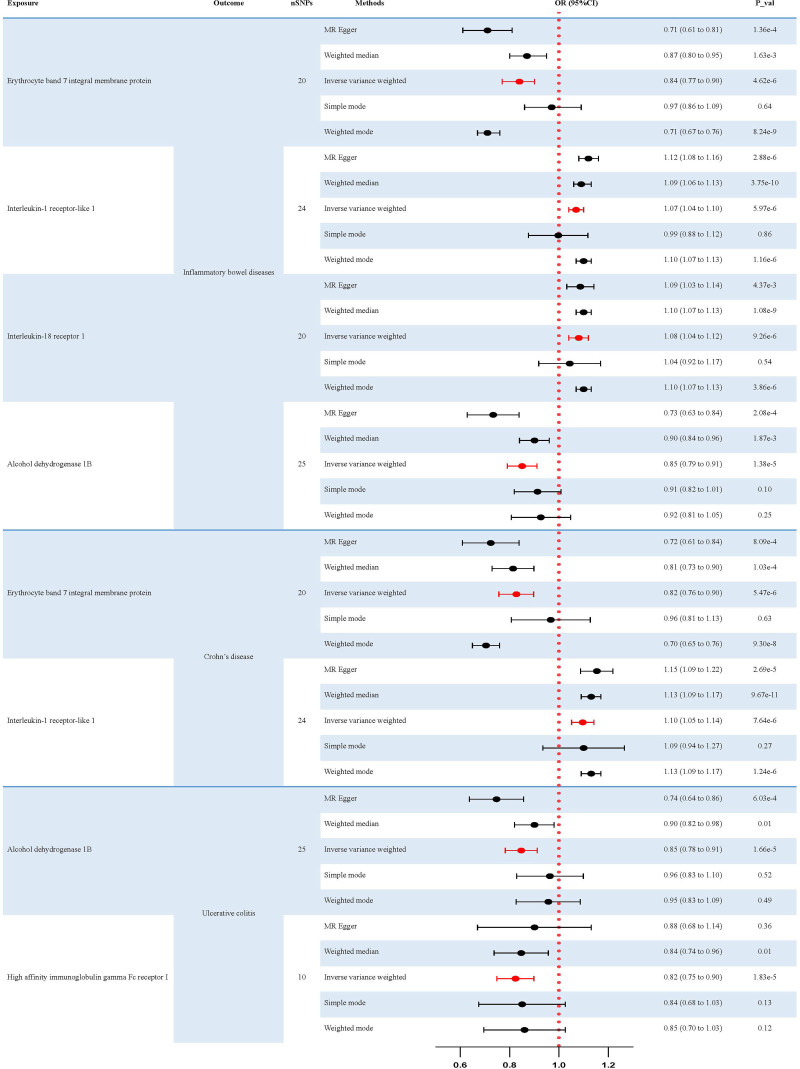
Results of two-sample MR analysis in evaluating the casual association between plasma protein phenotypes and IBD. MR = Mendelian randomization, IBD = inflammatory bowel disease.

**Figure 3.**
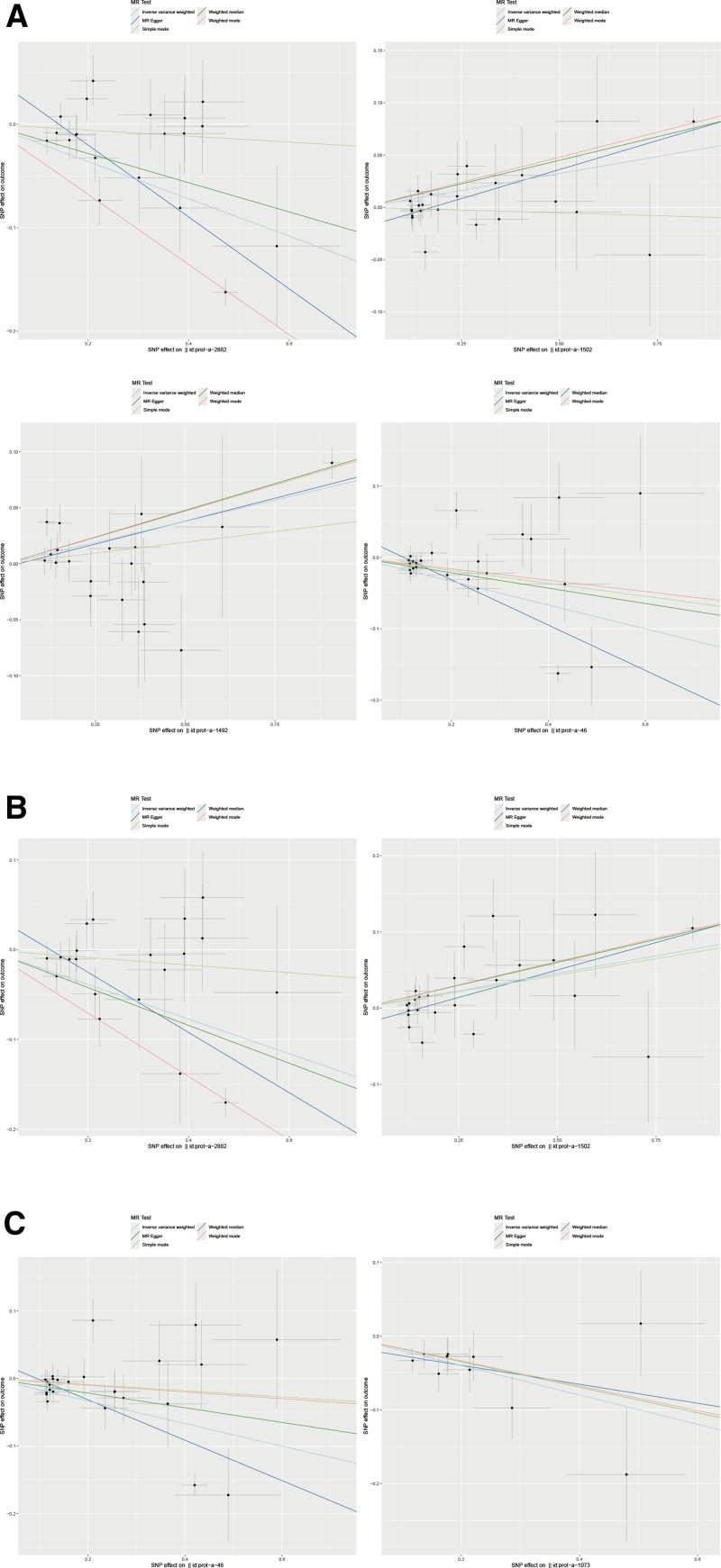
Scatter plots of causal relationships between plasma protein phenotypes and IBD, CD, and UC. (A) Scatter plots between plasma protein phenotypes and IBD. (B) Scatter plots between plasma protein phenotypes and CD. (C) Scatter plots between plasma protein phenotypes and UC. CD = Crohn disease, IBD = inflammatory bowel disease, UC = ulcerative colitis.

**Figure 4.**
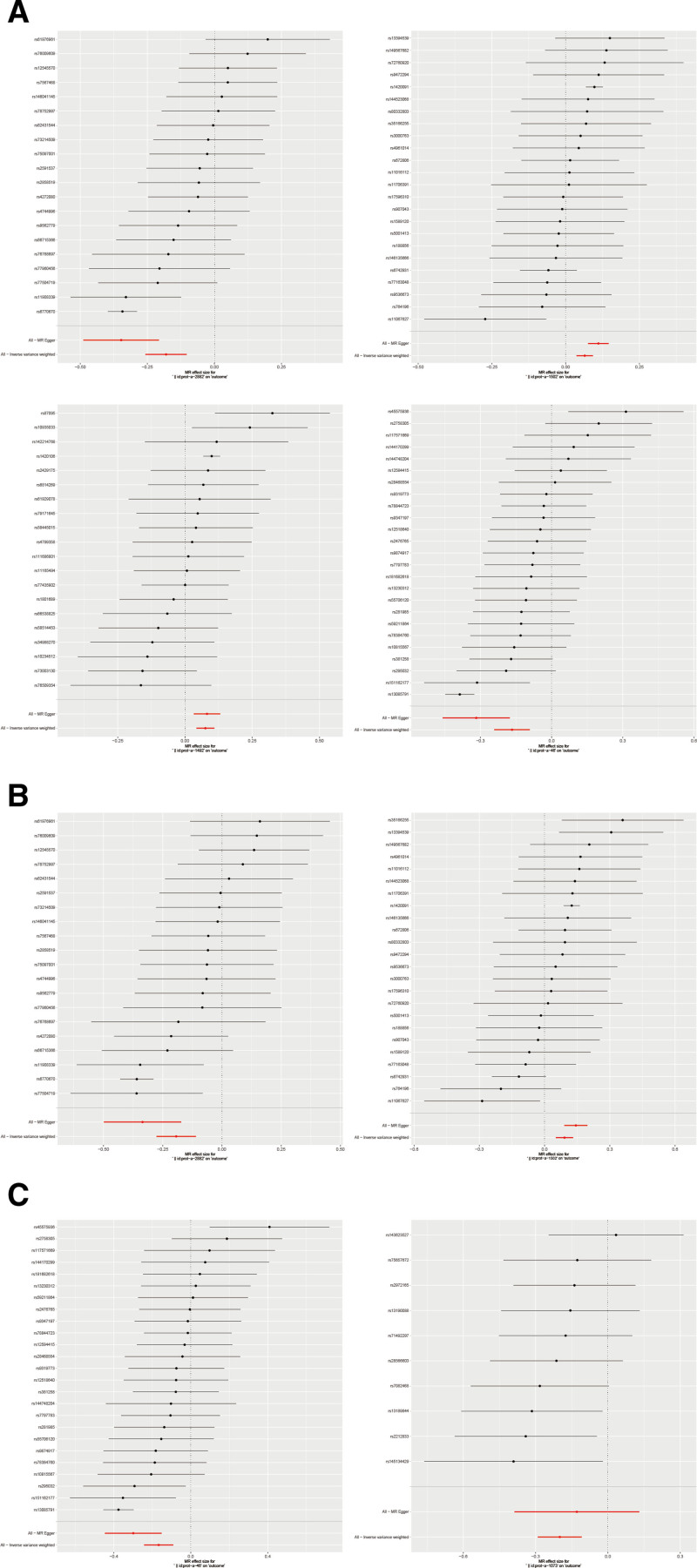
Forest plots of the strength of each IVs. (A) Forest plots of the IVs on IBD. (B) Forest plots of the IVs on CD. (C) Forest plots of the IVs on UC. CD = Crohn disease, IBD = inflammatory bowel disease, IVs = instrumental variables, UC = ulcerative colitis.

After Bonferroni correction, the *P*-values of plasma proteins affecting IBD and its histotypes (UC and CD) were below the Bonferroni threshold (Tables S8–S10, Supplemental Digital Content, https://links.lww.com/MD/Q147). To identify and screen plasma protein phenotypes that are associated with the risk of IBD, we performed a two-sample MR analysis using 3282 plasma protein phenotypes as exposures and IBD as outcomes (Tables S3–S5, Supplemental Digital Content, https://links.lww.com/MD/Q147). As shown in Tables S3–S5 (Supplemental Digital Content, https://links.lww.com/MD/Q147), we initially performed 5 methods of MR analysis to estimate linear regression. Subsequently, the IVW method of MR analysis was selected for the following calculations (Fig. 2). These results demonstrate that the 4 protein phenotypes have potential roles in the risk of IBD. Among them, Interleukin-1 receptor-like 1 and Interleukin-18 receptor 1 in the Interleukin-1 receptor family proteins are positively associated with the risk of IBD. In contrast, the levels of erythrocyte band 7 integral membrane protein and alcohol dehydrogenase 1 B showed negative relationships with the risk of IBD. The results of IVW analysis for the 4 types of plasma proteins are as follows: erythrocyte band 7 integral membrane protein (*P* = 4.62 × 10^−6^; OR 95% CI = 0.84 (0.77–0.90)), Interleukin-1 receptor-like 1 (*P* = 5.97 × 10^−6^; OR 95% CI = 1.07 (1.04–1.10)), Interleukin-18 receptor 1 (*P* = 9.26 × 10^−6^; OR 95% CI = 1.08 (1.04–1.12)), alcohol dehydrogenase 1B (*P* = 1.38 × 10^−5^; OR 95% CI = 0.85 (0.79–0.91)) (Fig. 2). The effect of erythrocyte band 7 integral membrane protein and Interleukin-1 receptor-like 1 was also observed in the Mendelian randomization analysis of the risk of CD (Table S4, Supplemental Digital Content, https://links.lww.com/MD/Q147). This trend is consistent with the influence of these 2 factors on IBD. The results of IVW analysis for the 2 types of plasma proteins are as follows: erythrocyte band 7 integral membrane protein (*P* = 5.47 × 10^−6^; OR 95% CI = 0.82 (0.76–0.90)), Interleukin-1 receptor-like 1 (*P* = 7.64 × 10^−6^; OR 95% CI = 1.10 (1.05–1.14)). Regarding the risk of UC (Table S5, Supplemental Digital Content, https://links.lww.com/MD/Q147), an effect of alcohol dehydrogenase 1 B was observed. At the same time, we also noted an inverse correlation between high-affinity immunoglobulin gamma Fc receptor I and disease risk. The results of IVW analysis for the 2 types of plasma proteins are as follows: Alcohol dehydrogenase 1B (*P* = 1.66 × 10^−5^; OR 95% CI = 0.85 (0.78–0.91)), High-affinity immunoglobulin gamma Fc receptor I (*P* = 1.83 × 10^−5^; OR 95% CI = 0.82 (0.75–0.90)). Scatter plots of causal relationships and forest plots of IVs are shown in Figures 3 and 4.

### 3.3. Results of statistical analysis

The results of Cochran *Q* test and leave-one-out method are shown in Table S6 (Supplemental Digital Content, https://links.lww.com/MD/Q147). Among them, it is worth mentioning that 3 SNPs (rs1420106, rs1420091, rs6770670) were found to have a large impact on the results. Sensitivity analysis using the MRegger and weighted median methods (Tables S3–S5, Supplemental Digital Content, https://links.lww.com/MD/Q147), we found that all plasma protein phenotypes that were significant in IVW were still significant in both methods, and had the same trend of influence on IBD and its histotypes. Unfortunately, the association between erythrocyte band 7 integral membrane protein and CD was not found when we excluded abnormal IVs leading to horizontal pleiotropy using the MRPRESSO method (Table S7, Supplemental Digital Content, https://links.lww.com/MD/Q147).

**Figure 5.**
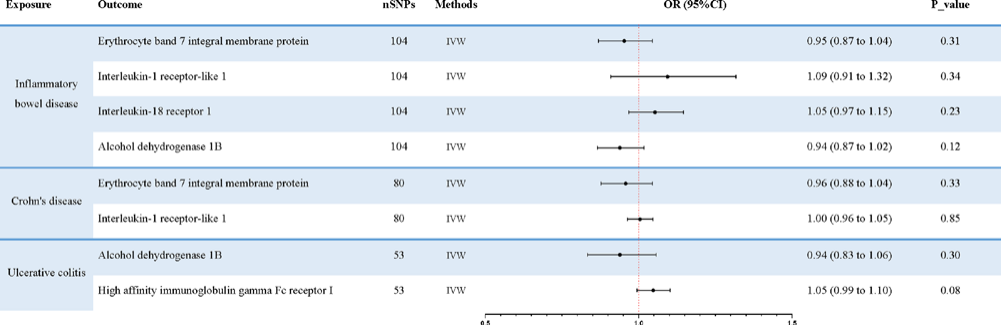
Results of two-sample MR analysis in evaluating the reverse casual association between plasma protein phenotypes and IBD. IBD = inflammatory bowel disease, MR = Mendelian randomization.

To further clarify the influence of plasma protein phenotypes on IBD and its subtypes, we performed reverse analysis of the significant results found in the previous step. Based on the reverse analysis results, we did not find a causal association between IBD and its subtypes among the 8 identified protein phenotypes (Fig. 5).

## 4. Discussion

This MR analysis identified substantial evidence indicating the influence of genetic predispositions to 4 plasma protein phenotypes in the development of IBD. MR analyses of histotypes indicated that 2 distinct protein phenotypes in both Crohn’s and ulcerative colitis are correlated with illness risk. This study is the first to investigate the correlation between the plasma protein phenotype and the status of IBD and its prevalent histotypes, employing an MR approach, which mitigates certain biases inherent in observational studies and may furnish data to substantiate causality.

Alongside the aforementioned Interleukin-1 (IL-1) receptor family proteins, we identified 3 plasma protein phenotypes linked to a diminished risk of IBD and its histotypes.^[23]^ Previous studies have shown that Interleukin-1 (IL-1) receptor family proteins are pivotal in modulating immune responses and inflammation, playing a crucial role in the pathogenesis of IBD, CD, and UC.^[24]^ For example, IL-1R1 mediates the action of IL-1β, a pro-inflammatory cytokine that is frequently upregulated in patients with IBD; IL-1RL1, or the receptor for IL-33, has gained attention in the context of IBD because of its dual role in promoting Th2 responses and contributing to inflammation; IL-18R1, the receptor for IL-18, also plays a significant role in the inflammatory processes associated with IBD. In addition to these receptors, other members of the IL-1 receptor family, such as IL-1RL2 (also known as the IL-36 receptor) and IL-1RAPL1, are gaining interest in the context of IBD. Specifically, IL-33 via IL-1RL1 activates Th2 cells, leading to the production of cytokines such as IL-5 and IL-13, which can exacerbate inflammation.^[25–28]^ This Th2-skewed response is particularly noted in UC, where mucosal integrity is often compromised, contributing to the chronic inflammation characteristic of the disease.^[29]^ The activation of IL-18R1 leads to downstream signaling that can exacerbate the inflammatory response, particularly in CD, where Th1 responses are predominant. In CD, the presence of IL-18 has been correlated with disease activity, indicating its potential as a biomarker of disease severity.^[30,31]^ Our MR Analysis results also proved the same trend, specifically demonstrating that genetically predicted higher levels of IL-1RL1 were associated with increased risk of IBD (OR = 1.07, 95% CI 1.04–1.10) and CD (OR = 1.10, 95% CI 1.05–1.14), while higher levels of IL-18R1 were associated with increased risk of IBD (OR = 1.08, 95% CI 1.04–1.12).

Nevertheless, the results of our MR Analysis were slightly different from those of previous studies. First, we found an effect of IL-1RL1 only in IBD and CD but not in UC. Second, regarding IL-18R1, we only found its role in IBD, and there was no significant correlation in the subtype analysis. Third, except for IL-1RL1 and IL-18R1, we did not find any correlation between other Interleukin-1 (IL-1) receptor family proteins and IBD or its histotypes. All of these factors need to be further explored and verified in follow-up research. These findings highlight the potential of IL-1RL1 and IL-18R1 not only as indicators of disease mechanisms but also as candidate biomarkers for specific IBD subsets (e.g., IL-1RL1 in CD) and potential therapeutic targets. Drugs modulating IL-33/ST2 (IL-1RL1) or IL-18 pathways are already in development for other inflammatory conditions, and our MR data supports exploring their application specifically in IBD and CD contexts.^[32]^

In addition to the Interleukin-1 receptor family proteins described above, we identified 3 plasma protein phenotypes associated with a reduced risk of IBD and its histotypes. Specifically, our MR analysis found that genetically predicted higher levels of erythrocyte band 7 integral membrane protein (BAND7) were associated with reduced risk of IBD (OR = 0.84, 95% CI 0.77–0.90) and CD (OR = 0.82, 95% CI 0.76–0.90), while higher levels of alcohol dehydrogenase 1B (ADH1B) were associated with reduced risk of IBD (OR = 0.85, 95% CI 0.79–0.91) and UC (OR = 0.85, 95% CI 0.78–0.91), and higher levels of High-affinity immunoglobulin gamma Fc receptor I (FcγRI/CD64) were associated with reduced risk of UC (OR = 0.82, 95% CI 0.75–0.90). These protective effect sizes warrant consideration for their clinical implications.

BAND7, is a member of the band 7 family of proteins that plays crucial roles in the maintenance of cell membrane integrity and function, particularly in erythrocytes.^[33]^ While research on BAND7 in the context of IBD is limited, emerging evidence suggests a connection between membrane proteins, erythrocyte function, and IBD pathogenesis. Patients with IBD often experience anemia, which is commonly attributed to chronic inflammation and malabsorption. Anemia can lead to changes in erythrocyte morphology and membrane characteristics. Alterations in the composition and function of erythrocyte membrane proteins, including BAND7, may affect the integrity and functionality of RBCs in these patients.^[34,35]^ These ideas are supported by our findings that BAND7 levels are inversely associated with the risk of IBD and CD. Further exploration of this relationship could enhance our understanding of IBD pathophysiology and lead to improved management strategies for anemia. The protective association identified by MR suggests BAND7 could be investigated as a potential biomarker reflecting erythrocyte health or resilience in IBD, potentially correlating with anemia severity or response to treatment.^[36]^ While direct therapeutic targeting may be challenging, understanding factors influencing BAND7 expression or function could inform strategies to improve erythrocyte stability in IBD patients.

Alcohol dehydrogenase 1B (ADH1B) is an enzyme involved in the metabolism of ethanol and other alcohols.^[37]^ In recent years, there has been increasing interest in the potential role of ADH1B in IBD, CD, and UC. Although the relationship between ADH1B and IBD is still being explored, current evidence suggests that ADH1B may influence the risk and progression of IBD through its role in alcohol metabolism, inflammatory response modulation, and interactions with gut microbiota.^[38]^ One plausible mechanistic link involves ADH1B’s role in acetaldehyde detoxification. Higher ADH1B activity, associated with specific genetic variants, leads to faster conversion of ethanol to acetaldehyde and subsequently acetate. This could reduce local ethanol concentrations in the gut, potentially mitigating ethanol-induced mucosal damage or alterations in gut permeability, factors implicated in IBD pathogenesis.^[39]^ Furthermore, ethanol metabolism significantly impacts gut microbiota composition and function. Slower metabolism (lower ADH1B activity) might allow more ethanol to reach the distal gut, promoting dysbiosis characterized by blooms of bacteria like Proteobacteria, which are often increased in active IBD.^[40]^ Higher ADH1B activity, by rapidly clearing ethanol, might help maintain a healthier microbial balance, thereby exerting a protective effect against IBD development, particularly UC which shows strong links to mucosal barrier dysfunction and dysbiosis.^[41]^ As research advances, a better understanding of this relationship may lead to improved management strategies for IBD patients. The MR evidence positions ADH1B activity or associated metabolites as potential biomarkers for IBD risk stratification. Therapeutically, strategies aimed at modulating local alcohol metabolism or targeting the downstream consequences of altered metabolism (e.g., acetaldehyde effects, microbial shifts) could be explored, although direct enzyme targeting presents challenges.

FcγRI, also known as CD64, is an important receptor that binds to the Fc region of IgG antibodies.^[42]^ Its involvement in inflammatory processes and immune regulation has made FcγRI a significant focus in the context of inflammatory diseases, including IBD, which comprises CD and UC. FcγRI is integral to regulating immune responses in the gut.^[43]^ In IBD, there is an imbalance in the immune system that leads to chronic inflammation. Studies have demonstrated that FcγRI facilitates the phagocytosis of IgG-coated antigens, which helps modulate local inflammatory responses.^[44]^ In IBD, especially UC, dysregulation of this process can contribute to ongoing inflammation and tissue damage.^[45]^ Unfortunately, in our study, we only found a protective effect of high-affinity immunoglobulin gamma Fc receptor I on UC. This specific protective effect for UC, contrasting with its known pro-inflammatory role upon activation, highlights the complexity of Fcγ receptor biology. Our finding of a genetically predicted higher level being protective might reflect a role in immune complex clearance or regulatory feedback mechanisms under homeostatic conditions, the disruption of which (e.g., saturation, altered ligand profiles in UC) could tip the balance towards inflammation.^[46]^ This contrasts with studies showing elevated FcγRI expression in inflamed UC mucosa, which likely reflects activation state rather than baseline genetically determined levels. Studies have shown that FcγRI expression is significantly elevated in the inflamed mucosal tissue of UC patients compared to healthy controls. This increased expression correlates with the severity of inflammation, suggesting that FcγRI is involved in perpetuating the inflammatory cycle in UC.^[47]^ The heightened expression of FcγRI on macrophages and neutrophils in UC patients may enhance their responsiveness to IgG-coated antigens, contributing to exaggerated immune responses in inflamed mucosa.^[45]^ These multidirectional results are likely related to the multiple roles of the immune response in the disease process. The discrepancy between our MR finding (protective association of genetically predicted levels) and observations of elevated expression in tissue (associated with inflammation) underscores the importance of context – baseline levels vs. induced expression during inflammation. Our MR result suggests that higher constitutive expression or activity of FcγRI might confer a protective homeostatic role in the gut, potentially through efficient clearance of immune complexes before they trigger inflammation.^[48]^ Its upregulation during active UC could then represent a consequence or amplifier of inflammation rather than the initiating cause. Therapeutically, this duality is important: while blocking FcγRI activation in established UC might be beneficial (as suggested by its inflammatory role), strategies aimed at enhancing its baseline clearance function could be explored for prevention or maintenance of remission.^[49]^ The specific UC association makes it a candidate biomarker for UC susceptibility or progression. Therefore, future research focusing on the mechanisms by which FcγRI influences immune responses in UC may provide new insights into the therapeutic approaches for managing this chronic inflammatory condition.

### 4.1. Study strengths

The present study has 2 main strengths. First, MR analysis is mainly based on the reuse of GWAS data, allowing re-analysis of existing GWAS data to explore potential causal associations between plasma protein phenotypes and IBD and its histotypes. Because of the random assignment of alleles during meiosis, MR studies are relatively immune to common behavioral, physiological, and socioeconomic confounders if all assumptions are met. In many cases, genetic variation is precisely measured and reported, and therefore, could be free of bias and error, which is particularly useful for assessing risk factors for long-term effects. Thus, MR is particularly informative for assessing the potential causal effects. Second, this study is the first to investigate potential causal associations between plasma protein phenotypes and IBD and its histotypes using MR methods. Such analyses leverage large GWAS data involving large numbers of disease cases and controls, which is an effective extension of the previous research.

### 4.2. Study limitations

The present study has several limitations. First, we assessed the relationship between plasma protein phenotypes and the risk of IBD and its histotypes, although the trends we observed were broadly the same across different approaches. However, there are differences in significance among the different analysis methods, and more analyses are not significant in the weighted and simple modes. Second, while we performed subtype-specific analyses for Crohn disease (CD) and ulcerative colitis (UC), these analyses had smaller sample sizes compared to the overall IBD analysis. This reduced statistical power may limit our ability to detect true associations, particularly for effects of smaller magnitude. Findings specific to CD or UC subtypes should therefore be interpreted with caution and require validation in larger, targeted studies. Third, our MR approach focuses exclusively on genetic associations. IBD is a multifactorial disease where environmental factors (e.g., diet, smoking, microbiome) play significant roles and may interact with genetic predispositions.^[50]^ Future studies integrating genetic data with comprehensive environmental exposure assessments are needed to provide a more holistic understanding of IBD etiology. Fourth, the present study only analyzed the associations from a genetic perspective using SNPs as IVs, and more in-depth exploration remains to be conducted. Fifth, the limitation of the study to populations of European descent reduces the potential for demographic bias but limits the generalizability of MR results to other populations.

## 5. Conclusion

In summary, using a comprehensive MR Study design, we found genetic evidence supporting a causal association between 5 plasma protein phenotypes and IBD and its common histotypes. The findings of this study provide valuable insight into the complex interactions between circulating plasma protein isoforms and IBD risk. These insights can inform collaboration among researchers to better monitor and screen for IBD and develop personalized medical strategies.

## Acknowledgments

We thank all the authors for their contributions to this study.

## Author contributions

**Conceptualization:** Zheng Jiao, Ge-Ge Li, Ling-Shuo Bai

**Data curation:** Zheng Jiao, Ge-Ge Li, Ling-Shuo Bai, Jian-Zeng Guo.

**Formal analysis:** Zheng Jiao, Ge-Ge Li, Ling-Shuo Bai, Jian-Zeng Guo.

**Funding acquisition:** Zheng Jiao.

**Methodology:** Zheng Jiao, Ge-Ge Li, Ling-Shuo Bai.

**Software:** Zheng Jiao, Ge-Ge Li, Ling-Shuo Bai, Jian-Zeng Guo.

**Supervision:** Zheng Jiao, Ge-Ge Li, Jian-Zeng Guo, Ling-Shuo Bai, Li-Jian Pang.

**Writing – original draft:** Zheng Jiao, Ge-Ge Li, Ling-Shuo Bai,Jian-Zeng Guo.

**Writing – review & editing:** Zheng Jiao, Ge-Ge Li, Jian-Zeng Guo, Ling-Shuo Bai, Li-Jian Pang.

## Supplementary Material


